# Mycolicibacterium mageritense Bacteremia Following Partial Nephrectomy: A Case Report

**DOI:** 10.7759/cureus.107482

**Published:** 2026-04-21

**Authors:** Robert Castro

**Affiliations:** 1 Division of Infectious Diseases, H. Lee Moffitt Cancer Center and Research Institute, Tampa, USA

**Keywords:** bacteremia, infectious disease medicine, non-tuberculous mycobacterium, postoperative fever, post-procedural bacteremia, rapidly growing mycobacterium

## Abstract

*Mycolicibacterium mageritense* is a rapidly growing non-tuberculous mycobacterium (NTM) that has been associated with multiple human infections, including surgical site, skin soft tissue, prosthetic joint, device-related, pulmonary infections, and bacteremia. The case reported is of a 74-year-old male who underwent partial nephrectomy for a renal mass and subsequently developed postoperative fever. Blood cultures revealed *Mycolicibacterium mageritense* bacteremia despite the absence of indwelling devices or central venous catheters. The patient was treated with a multidrug antibiotic regimen consisting of imipenem-cilastatin, cefoxitin, and ciprofloxacin, resulting in complete recovery. This case highlights that *Mycobacterium mageritense* bacteremia could be considered in cases of postoperative fevers without underlying hardware.

## Introduction

*Mycolicibacterium mageritense* is a rapidly growing non-tuberculous mycobacterium (NTM) that was first identified in 1997 [1]. It was previously called *Mycobacterium mageritense* until the name was changed in 2018, following a large phylogenomic study [2]. It is an environmental organism commonly found in the soil and water reservoirs [3]. It is closely related to *Mycobacterium fortuitum* and typically requires a multidrug antibiotic regimen for treatment [4]. Reported infections include post-procedural/post-surgical, primary skin and soft tissue infections, lymphadenitis, pulmonary infections, prosthetic joint infections, and device/catheter-related infections [5-18]. Among the reported cases with bacteremia, the majority involved the presence of central lines, cardiac implantable electronic devices (CIEDs), or, in one case, a left atrial pressure catheter [5,9,17]. There was one pediatric case who initially developed bacteremia in the setting of acute pancreatitis and without any reported long-term central line, hardware, or foreign material [7]. The case presented is of bacteremia in an adult patient following surgery, without an associated surgical site infection or hardware/central lines/foreign material. Treatment included a multidrug antibiotic regimen for six weeks, with resolution of the bacteremia and no post-treatment recurrence.

## Case presentation

A 74-year-old male with a history of a right renal mass, neurogenic bladder, diabetes mellitus type 2, Alzheimer’s dementia, and peripheral vascular disease initially presented for planned surgery for his right renal mass. On the day of admission, he underwent a complex right upper pole partial nephrectomy (pathology was consistent with renal cell carcinoma). He had no acute complaints leading up to his admission. He denied any fevers, chills, cough, shortness of breath, nausea, vomiting, abdominal pain, diarrhea, urinary changes, or new wounds or rashes. The day after surgery, he developed a fever. Vital signs included a temperature of 102.3°F, blood pressure of 180/105, respiratory rate of 16, and oxygen saturation of 97%. His physical exam revealed no acute distress, no rashes, normal rate and rhythm on cardiac examination, no lower extremity edema, lungs were clear to auscultation, his midline abdominal incision was intact and without erythema, and he was alert and oriented to person, place, and time.

**Figure 1:**
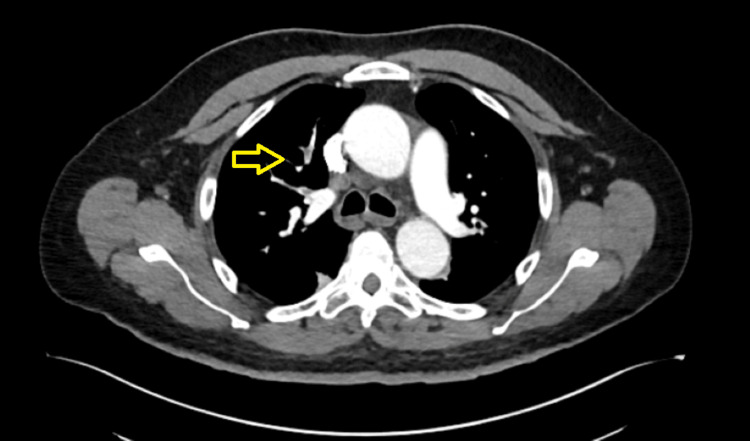
CT pulmonary angiography showing the right upper lobe pulmonary embolism.

**Figure 2:**
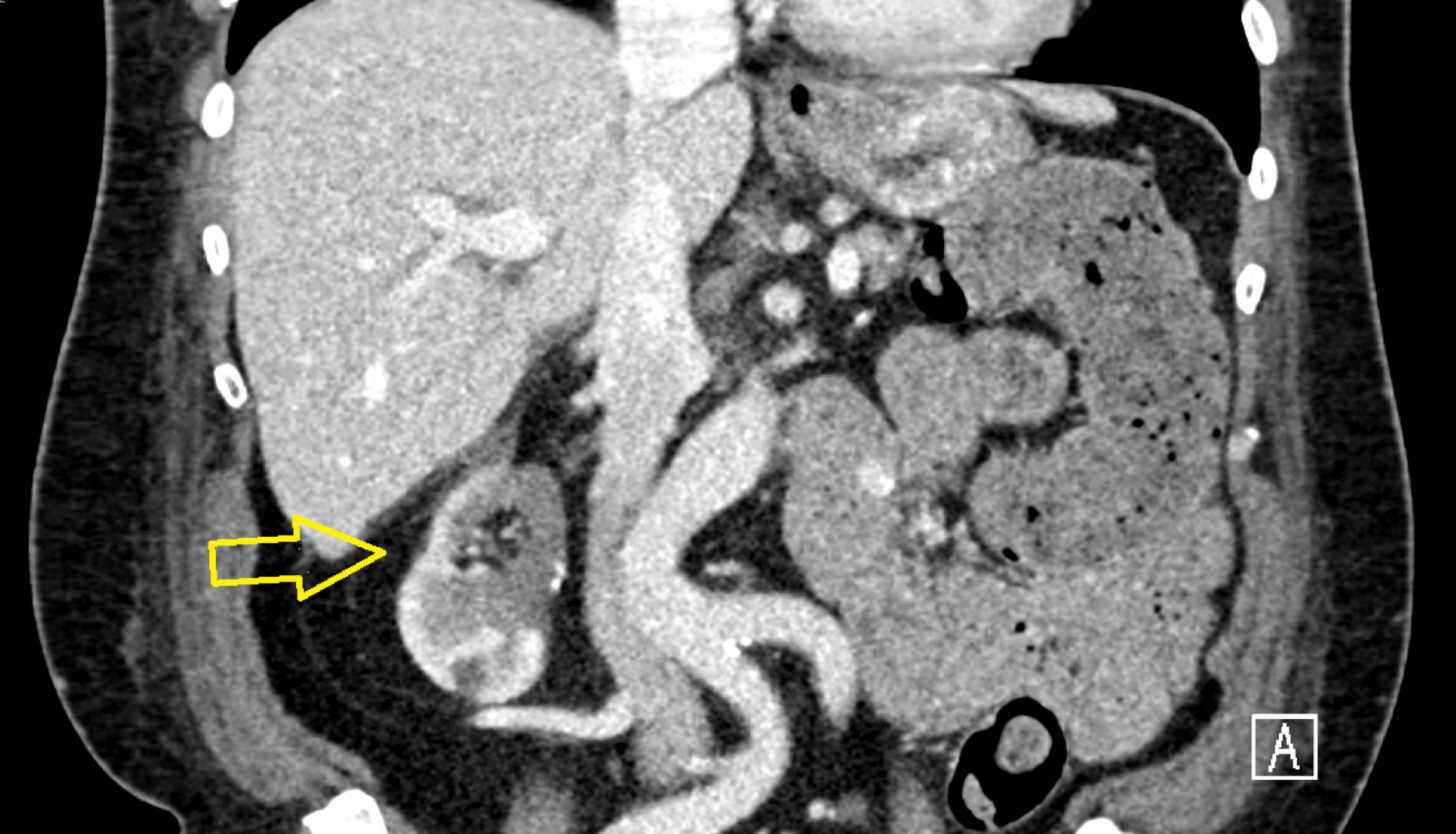
CT of the abdomen/pelvis showing the right partial nephrectomy site with postoperative changes and no abscess.

An infectious work-up was initiated, including blood cultures, urinalysis with reflex to urine culture, chest X-ray, methicillin-resistant *Staphylococcus aureus* (MRSA) nasal PCR screen, *Legionella* urine antigen, and *Streptococcus pneumoniae* urine antigen. Empiric antibiotics with ceftriaxone IV daily were started, which were then adjusted to cefepime IV every eight hours for broader empiric coverage. Blood cultures were repeated due to ongoing high fever (tmax = 103.1°F) 48 hours after initiation of IV antibiotics. His fevers resolved by postoperative day four. Two days after resolution of fever, the initial blood cultures taken when the fever started came back positive for a beaded gram-positive bacillus (acid-fast bacillus, AFB) in one of two peripheral sets. This organism was identified as *Mycolicibacterium mageritense*. Antibiotics were adjusted to imipenem-cilastatin IV, cefoxitin IV, and azithromycin oral (PO). To further assess the source of *Mycolicibacterium mageritense* bacteremia, a CT pulmonary angiography (PA) and CT of the abdomen/pelvis (A/P) with contrast were done. CT PA showed segmental thrombi in the right upper, middle, and lower lobe pulmonary arteries. The right upper lobe filling defects/pulmonary embolism are shown in Figure 1. CT A/P showed postoperative changes consistent with a partial right nephrectomy, with no well-defined abscess, as noted in Figure 2. A transthoracic echocardiogram (TTE) was performed as well, which was suggestive of a mitral valve vegetation, but a subsequent transesophageal echocardiogram (TEE) showed no evidence of endocarditis and a normal left ventricular ejection fraction. On identification of the organism, ciprofloxacin PO was added to the regimen, and azithromycin was discontinued, given previously reported susceptibility. He was discharged on an antibiotic regimen of imipenem-cilastatin IV, cefoxitin IV, and ciprofloxacin PO twice a day (BID) to complete a six-week course of antibiotics from the date of the last negative blood cultures. His labs during hospitalization and ultimate blood culture susceptibility results are presented in Table 1 and Table 2, respectively.

**Table 1:** Labs during hospitalization. WBC = white blood cells; Hgb = hemoglobin; PLT = platelets; Na = sodium; K = potassium; Cl = chloride; BUN = blood urea nitrogen; Cr = creatinine; Ca = calcium; Alk Phos = alkaline phosphatase; AST = aspartate aminotransferase; ALT = alanine aminotransferase; Mag = magnesium; HBV = hepatitis B virus; HCV = hepatitis C virus; QN = quantitative; sAg = surface antigen; (-) = no value present.

Description	Day 0	Day 1	Day 2	Day 6	Day 8	Day 12	Reference range and unit
WBC	7.38	9.46	5.04	3.7	4.84	6.21	(4-10.9) k/uL
Hgb	12.6	12.7	12.2	11.1	10.8	9.8	(13.4-16.9) g/dL
PLT	143	165	107	93	167	239	(143-382) k/uL
Na	143	142	144	140	143	139	(134-145) mmol/L
K	4.2	5	3.8	3.1	3.5	4	(3.4-4.5) mmol/L
Cl	109	109	113	107	106	106	(96-107) mmol/L
Glucose	192	167	185	158	117	138	(70-110) mg/dL
BUN	18	23	24	27	17	16	(6-23) mg/dL
Cr	0.8	1.6	1.3	1.2	1.1	1.2	(0.7-1.30) mg/dL
Ca	-	8	8.2	8.3	8.4	8.6	(8.6-10.2) mg/dL
Total protein	-	6.5	-	6.1	6.2	6.3	(6.2-8.4) g/dL
Albumin	-	3.9	-	3.1	3.1	3	(3.2-4.6) g/dL
Alk Phos	-	110	-	70	100	90	(40-130) U/L
AST	-	414	-	70	60	21	(11-34) U/L
ALT	-	279	-	100	77	25	(0-55) U/L
Mag	-	1.9	1.9	2.1	2.2	2.3	(1.6-2.3) mg/dL
Lactic acid	-	-	0.9	-	-	-	(0.5-2.0) mmol/L
Procalcitonin	-	-	0.42	-	-	-	(0-0.07) ng/mL
HCV IgG antibody	-	Reactive	-	-	-	-	Non-reactive or reactive
HCV QN PCR (blood)	-	-	Not detected	-	-	-	15-100,000,000 IU/mL QN range
HBV sAg (blood)	-	Non-reactive	-	-	-	-	Non-reactive or reactive
HBV QN PCR (blood)	-	-	Not detected	-	-		10-1,000,000,000 IU/mL QN range

**Table 2:** AFB blood culture susceptibility results. AFB = acid-fast bacillus; MIC = minimum inhibitory concentration; S = susceptible; I = intermediate; NI = no interpretation.

Mycolicibacterium mageritense		
Antibiotic	MIC	Interpretation
Amikacin	4	S
Cefoxitin	16	S
Ciprofloxacin	0.5	S
Clarithromycin	8	S
Clofazimine	<=0.03	NI
Doxycycline	2	I
Imipenem	0.5	S
Linezolid	<=1	S
Moxifloxacin	0.12	S
Trimethoprim/sulfa	20 c	S
Tigecycline	0.06	NI

He completed the antibiotics as planned. After five days post treatment, blood cultures (peripheral x 2) and AFB blood culture (x1) were repeated and were negative. He recovered without issues or complaints by the time his antibiotic course was completed.

## Discussion

The case presented was a postoperative infection presenting as isolated *Mycolicibacterium mageritense* bacteremia without an associated surgical site or distal site infection, in a patient without any underlying central line catheters, implanted devices, or prostheses. The reported cases of bacteremia caused by this organism in the literature have been associated with implanted devices or central venous catheters, except for one pediatric case, which presented with bacteremia and then later a subcutaneous abscess [5,7,9,17]. A postoperative fever prompted further infectious workup in this case. This is different in comparison to the cases reported by Tutzer et al., which presented as positive blood cultures initially in the setting of a retromammary abscess, Koyama et al., which presented as positive blood cultures in the setting of a local central venous access port infection, Fukunaga et al., which presented as positive blood cultures in the setting of a local CIED site infection, and Yamaguchi et al., which presented as positive blood cultures in the setting of a subcutaneous abscess [5,7,9,17]. The positive blood culture result with *Mycolicibacterium mageritense* was unexpected and required multiple antibiotic adjustments leading up to his eventual discharge. The variable susceptibility pattern, along with macrolide resistance, posed a challenge in treating this organism, necessitating a multidrug antibiotic regimen [1]. The duration of treatment is typically months long, with reported ranges of four weeks to one year, depending on the primary infection being treated (lymphadenitis, soft tissue infections, device-related infections, prosthetic joint infections, abscess) [5-19]. In this case, we treated with IV antibiotics alone for six weeks, without microbiologic or clinical recurrence. This case presentation had limitations, including the possibility of other etiologies contributing to his fevers (pulmonary embolism). Another limitation is the possibility of contamination or transient bacteremia, particularly as blood cultures cleared prior to targeted antimicrobial therapy. A possible reason for the blood culture discrepancy is that the repeat blood cultures performed in the hospital were only peripheral blood cultures, not dedicated AFB blood cultures. This case expands the clinical spectrum of *Mycolicibacterium mageritense* infections by demonstrating that transient postoperative bacteremia may occur even in the absence of a clear infectious focus or foreign body.

## Conclusions

Infections caused by *Mycolicibacterium mageritense* with associated bacteremia have been reported in adults with identifiable surgical site infection, primary infection, distal site of infection, or have been associated with underlying central line catheters, implanted devices, or prostheses. This case highlights the importance of considering *Mycolicibacterium mageritense *isolated bacteremia as a cause of postoperative fever and the importance of following up the blood culture susceptibility results to guide appropriate antibiotic therapy.
